# Femoral Head Osteonecrosis: Computed Tomography Not Needed to Identify Collapse When Using the Association Research Circulation Osseous Staging System

**DOI:** 10.1016/j.artd.2023.101244

**Published:** 2023-10-14

**Authors:** Eric Jordan, Nathan H. Varady, Shayan Hosseinzadeh, Stacy Smith, Antonia F. Chen, Michael Mont, Richard Iorio

**Affiliations:** a Department of Orthopaedic Surgery, Brigham and Women’s Hospital, Boston, MA, USA; b Department of Radiology, Brigham and Women’s Hospital, Boston, MA, USA; c Department of Orthopaedic Surgery, Rubin Institute for Advanced Ortho, Baltimore, MD, USA

**Keywords:** Osteonecrosis, ARCO staging, Hip, Femoral head, MRI, CT

## Abstract

**Background:**

The 2019 Revised Association Research Circulation Osseous (ARCO) Staging Criteria for Osteonecrosis of the Femoral Head (ONFH) only requires plain radiographs and magnetic resonance imaging (MRI) to diagnose and stage ONFH; however, the effectiveness of the 2019 ARCO criteria in the absence of computed tomography (CT) scans has not been investigated. Therefore, the purpose of this study was to evaluate whether CT scanning is a necessary modality for diagnosing/staging ONFH using the ARCO staging system. More specifically, do CT scans help differentiate pre- and post-collapse lesions more than MRI scans?

**Methods:**

A study was conducted on 228 ONFH patients diagnosed between January 1, 2008, and December 31, 2018, at a single academic medical center. CT and MRI scans were reviewed by the senior author and other contributors. The ONFH classification was compared between the 2 scans to determine if CT scans were able to further differentiate staging of collapsed lesions vs MRI scans.

**Results:**

A diagnosis of ONFH was made by MRI first in 57% (129/228) while 21% (48/228) used MRI and CT simultaneously. Only 22% (51/228) of cases were diagnosed by CT scans first. There were no cases where collapse was found by a CT scan that were not diagnosed by standard x-rays and/or MRIs.

**Conclusions:**

CT scans are not a useful adjunct for diagnosing or treating ONFH and are not necessary if MRI is ordered when using the Revised ARCO Staging System for ONFH diagnosis.

## Introduction

Osteonecrosis of the femoral head (ONFH) is a progressive condition that limits blood supply to bones, often leading to collapse of the femoral head and secondary osteoarthritis [1,2]. If left untreated, it can result in major, if not complete, loss of hip joint function, resulting in treatment by total hip arthroplasty (THA) in more than 70% of cases [3]. Approximately 10,000 to 20,000 new patients are diagnosed with ONFH each year, which accounts for up to 12% of the THA surgeries performed in the United States yearly [[4], [5], [6]]. Although severe ONFH may be treated by THA, early-stage ONFH can be treated using various surgical procedures such as osteotomy, bone grafting, or core decompression, which reduce intraosseous pressure and may induce bone healing, each of these procedures supplemented by various augmented marrow techniques [2].

A staging system has been developed to revise the 1994 Association Research Circulation Osseous (ARCO) Classification for ONFH [7]. The final consensus resulted in the following 4-tiered system: stage I—x-ray is normal, but either magnetic resonance imaging (MRI) or bone scan is positive; stage II—x-ray is abnormal (subtle signs of osteosclerosis, focal osteoporosis, or cystic change in the femoral head) but without any evidence of subchondral fracture, fracture in the necrotic portion, or flattening of the femoral head; stage III—fracture in the subchondral or necrotic zone as seen on the radiograph or computed tomography (CT) scans. This stage is further divided into stage IIIA (early, femoral head depression ≤2 mm) and stage IIIB (late, femoral head depression >2 mm); and stage IV—radiographic evidence of osteoarthritis with accompanying joint space narrowing, acetabular changes, and/or joint destruction.

ONFH is generally diagnosed by reviewing a patient’s medical history, and obtaining proper imaging assessments, to determine the stage of the disease [8]. Early detection and intervention are integral to preserving joint function. Although MRI accurately shows commonly seen alterations of the bone marrow in ONFH, CT also depicts areas of bony change [9]. The final consensus of the 2019 Revised ARCO classification for ONFH did not require CT for diagnosing and staging ONFH. Nevertheless, there are some investigators that believe that a CT scan is needed for accurate staging [[10], [11], [12], [13], [14]]. To our knowledge, no study has confirmed the utility of plain radiographs and MRI or evaluated the role of CT scans for the Revised ARCO diagnosis and staging of non-traumatic ONFH using a consecutive cohort of patients over a 10-year period. Therefore, the purpose of this study was to evaluate whether CT is a necessary modality for diagnosing and staging ONFH when using the Revised ARCO criteria. More specifically, were CT scans able to diagnose subchondral fractures or collapse, when standard x-rays or MRIs did not?

## Material and methods

Following institutional review board approval, a retrospective study was conducted including all consecutive patients who were diagnosed with ONFH from January 1, 2008, through December 31, 2018, from a single institution. Cases of diagnosed ONFH were identified using the following International Classification of Diseases-10 diagnosis codes: M87.05, M87.15, M87.25, M87.35, and M87.85. CT and MRI images of the hip, femur, and pelvis were thoroughly reviewed. Patients were only included if they had a diagnosis of ONFH and if they had both MRI and CT scans that evaluated ONFH.

The imaging modality leading to the initial diagnosis was identified for each patient. MRI scans performed after diagnosis of ONFH using CT in symptomatic patients were analyzed for staging utility relative to treatment decision. These imaging modalities were individually evaluated by the senior author to confirm diagnosis and concordance of interpretation between the musculoskeletal radiologist and the original treating surgeon. The involved hips were staged using the 2019 Revised ARCO Staging System [7].

All reported statistic values were presented with descriptive statistics. Excel (Microsoft, Redmond, WA) was utilized to compile these descriptive statistics. Student’s t-test was used to determine statistical significance.

## Results

**Figure 1:**
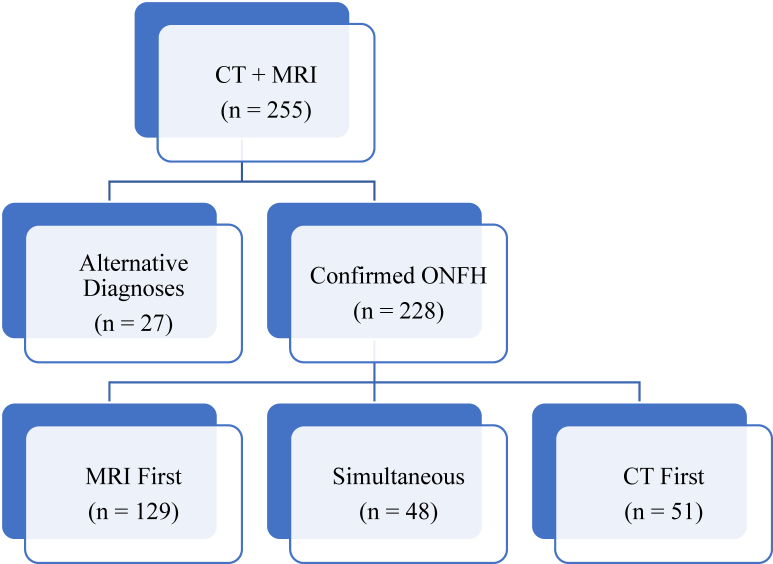
Flowchart of patient selection. Patients were excluded if they had not undergone both computed tomography (CT) and magnetic resonance imaging (MRI) or if a positive diagnosis of osteonecrosis of the femoral head (ONFH) was not confirmed following radiograph review.

There were 255 osteonecrosis patients who had both CT and MRI scans, with 228 (89%) having a definitive ONFH diagnosis by either CT and/or MRI and 27 (11%) yielding alternative diagnoses such as knee osteonecrosis. A diagnosis of ONFH was made by MRI first in 57% (129/228) of cases, while 21% (48/228) of patients were diagnosed with ONFH by MRI and CT simultaneously. There were 22% (51/228) of cases diagnosed by CT scan first (See Fig. 1). Ninety-four percent (48/51) of these CT-identified cases involved cancer (CA) diagnoses. The CT scans were used for CA staging and were not helpful with ARCO staging of ONFH. The other 3 cases identified asymptomatic ONFH.

Ninety-four percent (48/51) of these CT-identified cases involved CA diagnoses. The CT scans were used for CA staging and were not helpful with ARCO staging of ONFH. The other 3 CT scan cases were identified as asymptomatic ONFH. Of the 48 symptomatic ONFH cases identified by screening CT for CA, all received an MRI which led to accurate ARCO staging for treatment. There were no symptomatic ONFH cases which were not successfully staged and treated by MRI or required CT for management or treatment (*P* < .0001).

Of the 228 patients who had definitive ONFH diagnoses revealed by CT and/or MRI, 59 patients (26%) were found to have stage I disease, and 77 patients (34%) showed stage II disease. A total of 82 patients (36%) met the criteria for a stage III disease, with 52 patients (23%) being classified as stage IIIA and 30 patients (13%) as stage IIIB. Only 10 patients (4%) were placed in the category of stage IV disease.

There were no cases of stage III disease diagnosed by CT scan that were not diagnosed by standard x-rays and/or MRI scans.

## Discussion

To the best of our knowledge, no study has evaluated the necessity of CT scans for staging and diagnosing ONFH in accordance with the 2019 Revised ARCO Staging System. The results of this study suggest that CT scans are not a necessary imaging modality in cases of suspected ONFH. Despite having 228 patients who underwent both MRI and CT, the majority (78%) of ONFH diagnoses were made first by MRI or MRI and CT simultaneously. Only 22% (51/228) of the diagnoses were made first by CT scan alone. In addition, MRI was able to independently determine disease stage in these cases, where the diagnosis was made by CT scans.

These results demonstrate that ONFH patients undergoing an MRI scan can be staged accurately according to ARCO 2019 without undergoing a CT scan. This is important when considering the exposure to ionizing radiation and the financial burden of unnecessary CT scans [15,16]. This study confirms the findings of previous work evaluating the sensitivity of CT and MRI scans in diagnosing non-traumatic ONFH [[17], [18], [19], [20]] Our study also improved markedly on the size of the cohorts examined in these previous studies, which reviewed 18, 45, 49, and approximately 147 diseased hips, respectively [[17], [18], [19], [20]].

This study is potentially limited by biases due to the retrospective nature of the study. It is possible that CT scans performed for the purpose of ONFH diagnosis would be more accurate than survey CT scans. However, the ability of MRI to allow for accurate ARCO 2019 staging of all ONFH cases was definitive.

In summary, MRI was shown to be successful at detecting ONFH in our cohort. This imaging sufficiently staged both early- and late-stage ONFH using the 2019 Revised ARCO Staging System. MRI was useful for ARCO staging of ONFH and treatment decisions. Based on this retrospective study, CT scans are not necessary when using the Revised ARCO Staging System.

## Conflicts of interest

The authors declare there are no conflicts of interest.

For full disclosure statements refer to https://doi.org/10.1016/j.artd.2023.101244.

## Appendix A Supplementary data

Conflict of Interest Statement for Chen

Conflict of Interest Statement for Hosseinzadeh

Conflict of Interest Statement for Iorio

Conflict of Interest Statement for Jordan

Conflict of Interest Statement for Mont

Conflict of Interest Statement for Smith

Conflict of Interest Statement for Varady
