# Supplementary material for: Femoral Head Osteonecrosis: Computed Tomography Not Needed to Identify Collapse When Using the Association Research Circulation Osseous Staging System
Source: Arthroplast Today. 2023 Oct 14;24:101244. doi: 10.1016/j.artd.2023.101244 (PMC10585620; doi:10.1016/j.artd.2023.101244)
Supplement: Conflict of Interest Statement for Mont [file mmc5.pdf]

INDIVIDUAL CONFLICT OF INTEREST STATEMENT

American Association of Hip and Knee Surgeons

(Adopted from the American Academy of Orthopaedic Surgeons disclosure statement)

The following form **must be filled out completely and submitted by each author (example, 6 authors, 6 forms).**
All items require a response. If there is no relevant disclosure for a given item, enter "*None.*"

Manuscript Title: Femoral Head Osteonecrosis: Computed Tomography Not Needed to Identify Collapse When Using the Association Research Circulation Osseous (ARCO) Staging System

1. Royalties from a company or supplier (The following conflicts were disclosed)
Stryker

2. Speakers bureau/paid presentations for a company or supplier (The following conflicts were disclosed)
None

3A. Paid employee for a company or supplier (The following conflicts were disclosed)
None

3B. Paid consultant for a company or supplier (The following conflicts were disclosed)
Sage Products, Inc.; Stryker; TissueGene; 3M; Centrexion; CERAS Health; Flexion Therapeutics; Johnson & Johnson; Mirror-AR; NXSCI; Pacira; Peerwell; Pfizer-Lily; Skye Biologics; SOLVD Health; Smith & Nephew; US Medical Innovations

3C. Unpaid consultants for a company or supplier (The following conflicts were disclosed)
None

4. Stock or stock options in a company or supplier (The following conflicts were disclosed)
None

5. Research support from a company or supplier as a Principal Investigator (The following conflicts were disclosed)
National Institutes of Health

6. Other financial or material support from a company or supplier (The following conflicts were disclosed)
None

7. Royalties, financial or material support from publishers (The following conflicts were disclosed)
None

8. Medical/Orthopaedic publications editorial/governing board (The following conflicts were disclosed)
Journal of Arthroplasty Editor in Chief; Journal of Knee Surgery; Surgical Technology International; Orthopaedics

9. Board member/committee appointments for a society (The following conflicts were disclosed)
The Knee Society; The Hip Society

Each author must sign AND print or type his/her name, date and submit a separate form

In addition, one BLINDED Conflict of Interest form (no author names used) should be submitted per manuscript with all author disclosures.

Michael A. Mont, MD	Michael A. Mont	9/18/2022
Author Name (Print or Type)	Author Signature	Date
