# Supplementary material for: Femoral Head Osteonecrosis: Computed Tomography Not Needed to Identify Collapse When Using the Association Research Circulation Osseous Staging System
Source: Arthroplast Today. 2023 Oct 14;24:101244. doi: 10.1016/j.artd.2023.101244 (PMC10585620; doi:10.1016/j.artd.2023.101244)
Supplement: Conflict of Interest Statement for Smith [file mmc6.pdf]

INDIVIDUAL CONFLICT OF INTEREST STATEMENT

The Journal of Arthroplasty

(Adopted from the American Academy of Orthopaedic Surgeons disclosure statement)

The following form **must be filled out completely and submitted by each author (example, 6 authors, 6 forms)**. **If no discloser is required, please write/type "none" at the end of each sentence.**

Manuscript Title: Femoral Head Osteonecrosis: Computed Tomography Not Needed to Identify Collapse When Using the Association Research Circulation Osseous (ARCO) Staging System

1. Royalties from a company or supplier (The following conflicts were disclosed)
None

2. Speakers bureau/paid presentations for a company or supplier (The following conflicts were disclosed)
None

3A. Paid employee for a company or supplier (The following conflicts were disclosed)
None

3B. Paid consultant for a company or supplier (The following conflicts were disclosed)
None

3C. Unpaid consultants for a company or supplier (The following conflicts were disclosed)
None

4. Stock or stock options in a company or supplier (The following conflicts were disclosed)
None

5. Research support from a company or supplier as a Principal Investigator (The following conflicts were disclosed)
None

6. Other financial or material support from a company or supplier (The following conflicts were disclosed)
None

7. Royalties, financial or material support from publishers (The following conflicts were disclosed)
None

8. Medical/Orthopaedic publications editorial/governing board (The following conflicts were disclosed)
None

9. Board member/committee appointments for a society (The following conflicts were disclosed)
None

Each author must sign AND print or type his/her name, date and submit a separate form

In addition, one BLINDED Conflict of Interest form (no author names used) should be submitted per manuscript with all author disclosures.

Stacy E. Smith, MD	Stacy E. Smith, MD	10/2/22
Author Name (Print or Type)	Author Signature	Date
